# Evaluation of Macular and Peripapillary Blood Flow in Response to Intraocular Pressure Reduction in Patients With Posner–Schlossman Syndrome

**DOI:** 10.3389/fphys.2022.886871

**Published:** 2022-06-01

**Authors:** Dan Liu, Cong Fan, Endong Zhang, Jie Yang, Yue Zhang, Jian Jiang

**Affiliations:** ^1^ Eye Center of Xiangya Hospital, Central South University, Changsha, China; ^2^ Hunan Key Laboratory of Ophthalmology, Changsha, China; ^3^ National Clinical Research Center for Geriatric Disorders, Xiangya Hospital, Central South University, Changsha, China

**Keywords:** OCTA, PSS, peripapillary microcirculation, macular microcirculation, IOP reduction

## Abstract

**Purpose:** The study aimed to evaluate the effect of intraocular pressure (IOP) reduction on macular and peripapillary microcirculation in patients with Posner–Schlossman syndrome (PSS) by optical coherence tomography angiography (OCTA).

**Methods:** A prospective comparative study was conducted. Patients diagnosed with PSS at the Eye Center of Xiangya Hospital, Central South University, from February 2020 to November 2021 were consecutively included. OCTA was used for the macular and peripapillary microcirculation measurements, and optical coherence tomography (OCT) was employed for the retinal nerve fiber layer (RNFL) and lamina cribrosa depth (LCD) measurements. The patients received OCT and OCTA examinations at baseline and 1 week post-treatment when the IOP was under control. Changes in macular and peripapillary microcirculation, RNFL, and LCD were calculated for all the analyzed areas.

**Results:** Twenty-one eyes from 21 patients were included in the study. Pre-treatment and post-treatment IOP were 43.17 ± 10.36 mm Hg (range, 30–60 mm Hg) and 17.17 ± 2.85 mm Hg (range, 13–23 mm Hg), respectively. No statistically significant changes were detected in RNFL, LCD, or macular and peripapillary microcirculation after significant IOP reduction.

**Conclusion:** The results suggested that a large IOP reduction may not result in a significant increase in peripapillary and macular capillary perfusion in patients with PSS.

## Highlights

1. Up until now, how the changes in intraocular pressure (IOP) affect the retinal blood flow has not been completely demonstrated.
2. IOP reduction might not result in a significant increase in peripapillary and macular capillary perfusion in patients with Posner–Schlossman syndrome (PSS).
3. The defects in the neural structure of the patients, such as lamina cribrosa (LC) displacement and retinal nerve fiber layer (RNFL) thinning, but not changes in IOP itself, may be more responsible for the changes in the retinal blood flow.

## Introduction

Elevated intraocular pressure (IOP) is the major risk factor for glaucoma, and IOP reduction is the only proven way to slow down the disease progression (Leske et al., 2003) (Heijl et al., 2002). In addition to elevated IOP, there is a growing body of evidence indicating that reduced perfusion of the optic nerve head (ONH) and retina is positively associated with the progression of glaucoma (Yanagi et al., 2011; Liu et al., 2015; Wang et al., 2015). The blood perfusion of the ONH and retina depends on the balance between the local arterial blood pressure and IOP, which means that the elevation of IOP or the reduction of the arterial blood pressure may lead to a reduction in retinal blood circulation (Zheng et al., 2010; Baek et al., 2019). However, complex regulatory mechanisms are involved in ONH and retinal microcirculation, which can maintain the blood flow over large alterations in blood pressure or IOP (Schmidl et al., 2012; Boltz et al., 2013; Guidoboni et al., 2014). So far, it has not been completely demonstrated how the IOP changes affect the retinal blood flow. Previous studies have reported controversial findings about the effect of IOP reduction on ocular blood flow in glaucomatous eyes. Specifically, some studies showed a significant increase in vessel densities after IOP reduction (either medically or surgically) (Holló, 2017; Shin et al., 2017; In et al., 2018), while other studies found no change in blood flow after IOP reduction (Cantor, 2001; Kurvinen et al., 2014; Zéboulon et al., 2017).

Observation of hemodynamic changes in macular and peripapillary circulation in response to changes in IOP may provide some clues to understanding the pathogenesis of glaucoma. Since its introduction in 2012, optical coherence tomography angiography (OCTA) has shown better performance in the quantitative assessment of retinal vasculature than conventional angiography. It can provide noninvasive, rapid, detailed, reproducible, quantitative information about the microcirculation of ONH and retina (Moghimi et al., 2019; Werner and Shen, 2019; Rao et al., 2020). Therefore, in this study, we evaluated the changes in retinal microcirculation by OCTA.

Currently, the scleral suction cup (Pillunat et al., 1997; Nagel and Vilser, 2004), darkroom prone provocative test (Zhang et al., 2018), and laser peripheral iridotomy (LPI) (Ma et al., 2019; Wang et al., 2020) are the commonly used models of elevated IOP to study the effect of IOP change on retinal vascularization. But the results of these studies are also confusing. Some showed the retinal microcirculation was not related to the change in IOP, while others showed that retinal vessels may change at a critical IOP level, and the critical IOP level was different in different studies. (Pillunat et al., 1997; Nagel and Vilser, 2004; Zhang et al., 2018; Ma et al., 2019; Wang et al., 2020). The controversial data were attributed to the following shortcomings in these models. First of all, the aqueous flare and small pupil post-LPI treatment will compromise the quality of the OCTA examination. Second, the elevated IOP was time-sensitive by scleral suction cup and LPI so that the time window for the OCTA examination may not be long enough, which consequently weakens the stability of the OCTA examination. Last but not least, in the darkroom prone provocative test and LPI, the elevated IOP was not high enough, which was less than 40 mmHg. However, the IOP level was usually over 40 mmHg in many glaucomatous cases. To overcome the aforementioned limitations in previous models, Posner–Schlossman syndrome (PSS) was adopted as a natural model of high IOP in our study.

PSS is a special type of glaucoma that is characterized by recurrent, acute attacks of mild, unilateral, nongranulomatous anterior uveitis accompanied by markedly elevated IOP. During the acute attacks, IOP is always markedly high (the mean IOP in published studies varies from 42.77 to 49.2 mm Hg), and the high IOP can remain for several days (Megaw and Agarwal, 2017). Continuously high IOP can simulate the IOP of glaucoma and can also ensure the stability of IOP during an OCTA examination. In addition, the function and structure of the retina and optic nerve in patients with PSS are normal, which can rule out the changes in the retinal microvasculature caused by optic and retinal structural damage. Therefore, we investigated the effect of IOP reduction on ONH and retinal microcirculation in PSS patients. The purpose of this study was to clarify how retinal microcirculation changes in response to a large IOP reduction.

## Methods

### Patients

**FIGURE 1:**
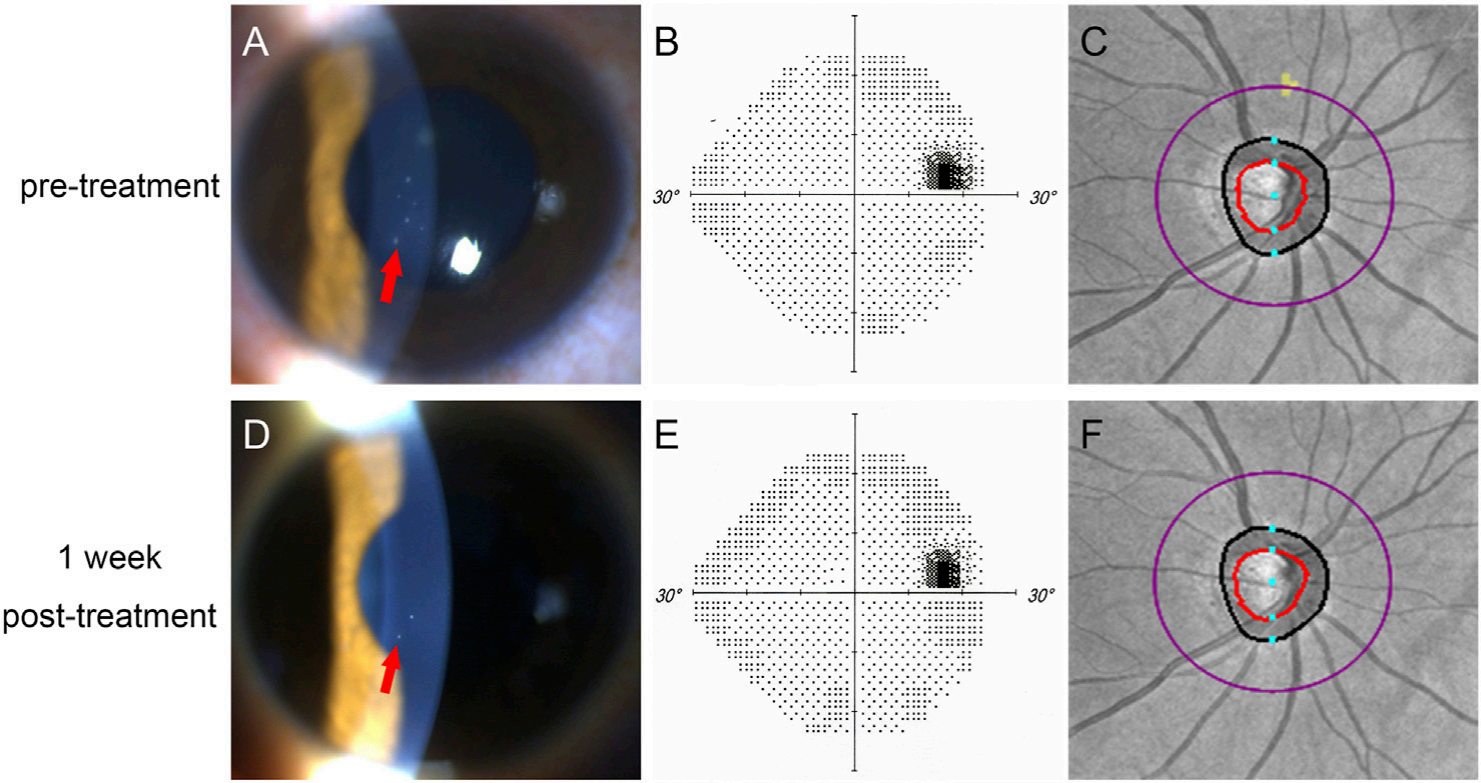
One patient in our study. **(A–C)**: before treatment; **(D–E)**: 1 week post-treatment. **(A,C)** showed that KP (red arrow) was reduced after treatment; **(B,D)** showed normal visual field before and after treatment; **(C,E)** showed that normal retinal nerve fiber layer before and after treament.

A prospective comparative study was conducted at the Eye Center of Xiangya Hospital, Central South University, from February 2020 to November 2021. The clinical study registration number was ChiCTR2100050912. A total of 21 patients diagnosed with PSS were consecutively included. The diagnostic criteria for PSS were as follows: 1) transient episode of elevated IOP; 2) mild anterior chamber inflammation and/or a few small-to-medium, discrete, round, white keratic precipitates (KP), accumulating in the lower half of the cornea, without iris posterior synechiae (Figure 1); 3) deep anterior chamber with a wide and open angle; and 4) normal visual field (VF), normal retinal nerve fiber layer (RNFL), and normal optic disc structure (Figure 1). The exclusion criteria were as follows: 1) the use of systemic or topical medication that could affect ocular circulation; 2) diabetes mellitus; 3) a history of prior intraocular surgery or ocular trauma; 4) any macular disease; 5) serious corneal edema or cataract that might affect the quality of fundus images; 6) the inability to cooperate; 7) IOP at the initial visit below 30 mm Hg; and 8) systemic hypertension with uncontrolled blood pressure. The study was approved by the Ethical Review Committee of Xiangya Hospital, Central South University, China, and it adhered to the provisions of the Declaration of Helsinki for research involving human participants. Written informed consent was obtained from all of the participants involved in the study. The patients’ unaffected eyes were included in the control group. If both eyes were affected, only the eye with the higher IOP was included in the affected group.

### Ophthalmologic Examination and Treatment

At the initial visit, all of the subjects underwent a comprehensive ophthalmologic examination, including best corrected visual acuity (BCVA), slit-lamp examination, gonioscopy, IOP measured by Goldmann applanation tonometry, fundus examination, axial length measurement (IOL Master, Carl Zeiss Meditec, Dublin, CA, United States), VF test (Humphrey Field Analyzer with Swedish Interactive Threshold Algorithm standard 24-2 test; Carl Zeiss Meditec, Dublin, CA, United States), spectral domain (SD)-OCT (Carl Zeiss Meditec, Dublin, CA, United States), and blood pressure examination. A history of smoking, diabetes, hypertension, and the use of systemic and ocular medications were recorded. After the initial evaluation, 0.01% prednisolone (four times a day), 0.5% timolol (twice a day), and 0.2% brimonidine eye drops (twice a day) were given to control the IOP. At 1-week follow-up, BCVA, slit-lamp examination, fundus examination, IOP measurement, VF test, OCT, and OCTA examination were performed. The blood pressure was measured right before the OCTA examination.

### Measurement of the Lamina Cribrosa Depth

**FIGURE 2:**
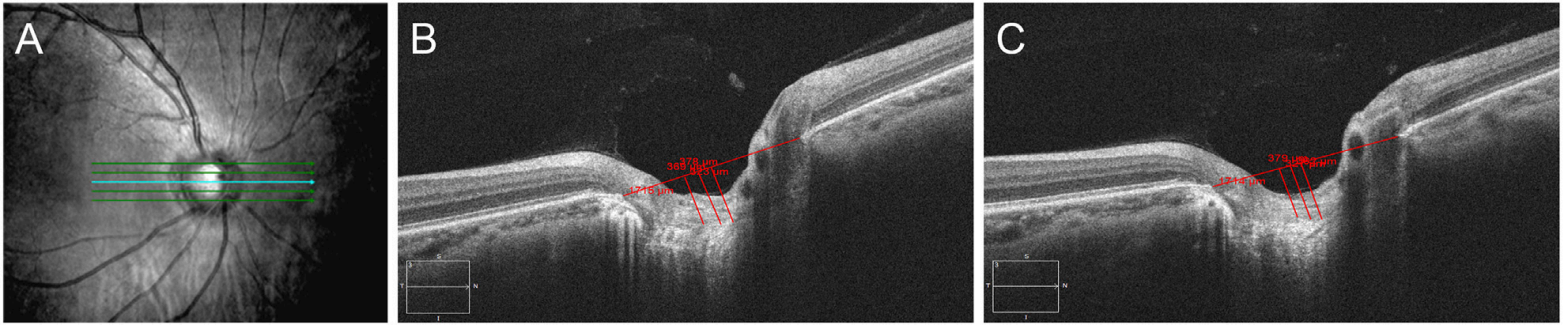
Measurement of LCD by SD-OCT. **(A)**: Five B-scan images were spaced equidistantly across the vertical optic disc diameter; **(B,C)**: LCD was measured at three points, including the maximum depth point and two additional points (100 and 200 μm away from the maximum depth point in the temporal direction). **(B)**: pre-treatment; **(C)**: 1 week post-treatment.

LCD was measured with SD-OCT (Carl Zeiss Meditec, Dublin, CA, United States) at the initial visit and at the 1-week follow-up. To determine LCD, five B-scans were selected from the three-dimensional image dataset of SD-OCT (Seo et al., 2014). The five B-scan images were spaced equidistantly across the vertical optic disc diameter. A line connecting the Bruch’s membrane edges was used as a reference plane, and LCD was measured in the perpendicular direction from the reference plane to the anterior surface of the lamina cribrosa (LC). LCD was measured at three points, including the maximum depth point and two additional points (100 and 200 μm away from the maximum depth point in the temporal direction). Only the temporally adjacent points were selected because the maximally depressed point was often close to the central vessel trunk, which had a shadow that obscured the LC (Figure 2). The average of the three measurements was taken as the LCD of each plane. The average of the LCDs of the five planes was defined as the mean LCD of the eye (Figure 2).

### Optical Coherence Tomography Angiography

All of the subjects underwent SD-OCTA examinations using CIRRUS AngioPlex 5000 equipment (Carl Zeiss Meditec, Dublin, CA, United States). Two OCTA scans of the macula and papilla were performed consecutively with a 1 min interval between the tests. These examinations were performed by a trained ophthalmologist (J.Y.) in a masked manner without knowledge of the results of the other examinations.

**FIGURE 3:**
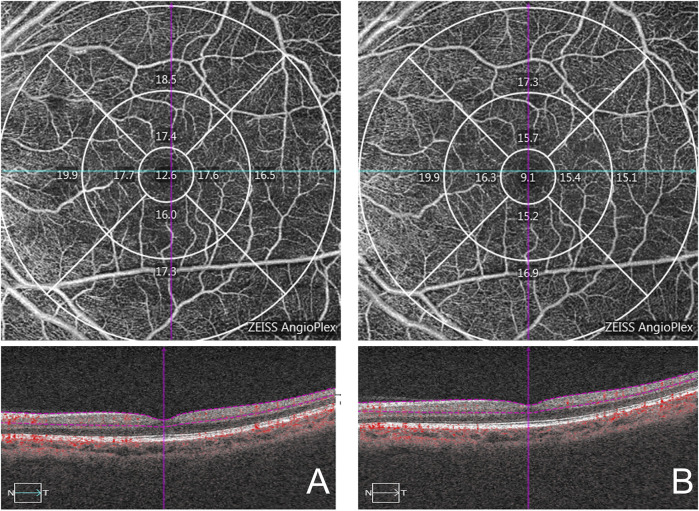
Macular area measurement map. The central area is the center 1 mm diameter range, the inner ring and the outer ring are 3 mm, 6 mm diameter range respectively. **(A)**: pre-treatment; **(B)**: 1 week post-treatment.

At the macula, a 6 mm × 6 mm volumetric macular superficial retinal vessel image that consisted of vessels from the layer of the inner limiting membrane (ILM) to the inner plexiform layer (IPL) was obtained. The system software divided macular images into three regions, namely, a central circle (1 mm radius from the fovea), an inner circle (1–3 mm radius from the fovea), and an external circle (3–6 mm radius from the fovea) (Figure 3). The parameters analyzed by AngioPlex^TM^ (software version 10.0.0.14618) were the macular vascular length density (VLD; defined as the total length of the perfused vasculature) and vessel perfusion density (VPD; defined as the total area of the perfused vasculature per unit area in a region of measurement).

**FIGURE 4:**
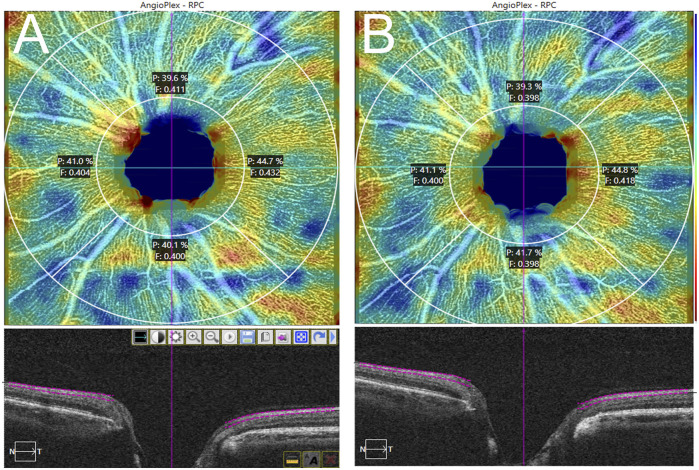
Radial peripapillary capillary plexus 4.5 × 4.5 mm scan. Plexus segmentation and analysis by AngioPlex^TM^. Signal strength: 10/10. **(A)**: pre-treatment; **(B)**: 1 week post-treatment.

Regarding the ONH, a 4.5 mm × 4.5 mm volumetric scan centering on the optic nerve was used. AngioPlex^TM^ automatically segmented a ring (region of interest; ROI) limited by an internal and external radius of 2.0 and 4.5 mm, respectively, which was, in turn, subdivided into four quadrants (superior, temporal, inferior, and nasal) (Figure 4). For this ROI, the capillary perfusion (CP; defined as the percentage of the area that contains the perfused vasculature in the ROI, expressed in %) and the flux index (FI; defined as the capillary perfusion, weighted by the brightness of the flow signal, where FI values range from 0 to 1) were measured.

Poor quality scans with signal strength index (SSI) values of less than 8 were excluded from the study.

### Statistical Analysis

Statistical analyses were performed in SPSS software (SPSS for Windows, v. 20.0; SPSS, Inc., Chicago, IL, United States). Data were represented as mean ± standard deviation. Measurements obtained before and after treatment were compared using the paired *t*-test. Differences were considered to be significant if *p* values were lower than 0.05.

## Results

### Clinical Features and Parameters of the Participants

Twenty-one patients were included in this study, and two of them had both eyes affected. Therefore, 21 affected eyes and 19 unaffected eyes were included in this study. Table 1 summarizes the clinical data of all of the subjects. Because most of the recurrent patients had already been treated with medications when they came to our clinic, the majority of the recurrent patients were excluded. In our study, 18 patients had their first attack; two patients had a history of two attacks within a year; and one patient had a history of three attacks within two years. Fifteen out of 21 patients had had eye pain for one day before they went to see an ophthalmologist. The mean untreated IOP was 43.17 ± 10.36 mm Hg (range, 30–60 mm Hg). Among these 21 patients, the untreated baseline IOP was more than 50 mm Hg in nine patients. All of the patients were very sensitive to medication treatment, and the duration of high IOP was 1.52 ± 0.87 days. In the first week post-treatment, the IOP of 20 patients was reduced to a normal level, and the mean IOP was 17.17 ± 2.85 mm Hg (range, 13–23 mm Hg). IOP was decreased significantly (*p* < 0.001), and the mean reduction of IOP was 26.05 ± 7.91 mm Hg (60.3%). The number of KP reduced, and BCVA improved significantly (*p* < 0.001), but the RNFL thickness, VF, LCD, and blood pressure did not change significantly (*p* = 0.73, *p* = 0.68, *p* = 0.45, *p* = 0.34, and *p* = 0.44, respectively) (Figures 1, 2).

**TABLE 1:** Clinical features and parameters of the participants.

	Affected eye		Unaffected eye	Pre-treatment vs. post-treatment	Affected eye vs. unaffected eye
	Pre-treatment	Post-treatment			
Age (years)	41.82 ± 7.20 (range, 34–49 years)			—	—
Sex (F/M)	9/12			—	—
Duration (days)	1.52 ± 0.87		—	—	—
AL (mm)	23.98 ± 0.67		23.79 ± 0.78	—	0.89
Number	21		19	—	—
Mean IOP (mm Hg)	43.17 ± 10.36 (30–60)	17.17 ± 2.85 (13–23)	16.34 ± 3.34	<0.001	<0.001
BCVA (LogMAR)	0.22 ± 0.16	−0.06 ± 0.04	−0.06 ± 0.03	<0.001	<0.001
RNFL thickness (μm)	98.00 ± 9.80	99.32 ± 9.50	99.54 ± 8.50	0.73	0.78
VF MD (DB)	−0.98 ± 0.72	−0.93 ±0 .0.62	−0.79 ± 0.53	0.68	0.72
LCD (μm)	395.45 ± 24.12	394.32 ± 23.15	393.15 ± 22.11	0.45	0.12
SSI	9.23 ± 1.01	9.64 ± 0.67	9.65 ± 0.57	0.27	0.31
SBP (mm Hg)	126.34 ± 16.23	128.12 ± 19.20	—	0.34	—
DBP (mm Hg)	80.79 ± 14.68	82.36 ± 16.38	—	0.44	—

AL, axial length, IOP, intraocular pressure, BCVA, best corrected visual acuity, LCD, lamina cribrosa depth, RNFL, retina nerve fiber layer, VFMD, visual field mean deviation, SSI, signal strength index, SBP, systolic blood pressure, DBP, diastolic blood pressure.

### Macular and Peripapillary OCTA Parameters of the Participants

Tables 2, 3 show the macular and peripapillary OCTA parameters in patients with PSS. Compared with the unaffected eyes, the affected eyes’ baseline VLD and VPD values in the central, inner, outer, and full macular regions were not significantly different; likewise, there were no significant differences in CP and FI values in the superior, inferior, nasal, temporal, and whole peripapillary areas between the affected and unaffected eyes (Figures 3, 4). After 1 week of being treated with medication, the affected eyes’ VLD and VPD values in the central, inner, outer, and full macular regions did not significantly change compared with the baseline; likewise, there were no significant changes in CP and FI values in the superior, inferior, nasal, temporal, and whole peripapillary areas.

**TABLE 2:** Macular OCTA parameters of the participants before and after treatment.

	VLD					VPD				
	Affected eye		Unaffected eye	Pre-treatment vs. post-treatment	Affected vs. unaffected	Affected eye		Unaffected eye	Pre-treatment vs. post-treatment	Affected vs. unaffected
	Pre-treatment	Post-treatment				Pre-treatment	Post-treatment			
Central	6.42 ± 3.37	6.46 ± 2.59	6.45 ± 2.41	0.97	0.87	0.14 ± 0.08	0.14 ± 0.04	0.14 ± 0.05	0.97	0.87
Inner	15.65 ± 3.14	16.41 ± 1.93	16.39 ± 1.87	0.46	0.43	0.37 ± 0.08	0.38 ± 0.05	0.38 ± 0.06	0.61	0.65
Outer	16.92 ± 2.10	17.34 ± 1.69	17.40 ± 1.78	0.58	0.54	0.41 ± 0.06	0.43 ± 0.05	0.43 ± 0.04	0.51	0.54
Full	16.33 ± 2.29	16.79 ± 1.61	16.80 ± 1.72	0.56	0.61	0.42 ± 0.04	0.41 ± 0.04	0.42 ± 0.03	0.61	0.68

VLD, vascular length density; VPD, vessel perfusion density.

**TABLE 3:** Peripapillary OCTA parameters of the participants before and after treatment.

	CP (%)					FI				
	Affected eye		Unaffected eye	Pre-treatment vs. post-Treatment	Affected vs. unaffected	Affected eye		Unaffected eye	Pre-treatment vs. post-Treatment	Affected vs. unaffected
	Pre-treatment	Post-treatment				Pre-treatment	Post-treatment			
Superior	42.79 ± 3.09	43.38 ± 2.69	43.26 ± 2.92	0.37	0.41	0.43 ± 0.03	0.44 ± 0.02	0.44 ± 0.03	0.69	0.98
Inferior	44.34 ± 2.91	44.23 ± 1.67	44.35 ± 2.61	0.83	0.93	0.43 ± 0.03	0.43 ± 0.02	0.43 ± 0.02	0.80	0.76
Nasal	42.93 ± 1.99	42.86 ± 1.77	42.91 ± 1.19	0.87	0.23	0.44 ± 0.04	0.43 ± 0.03	0.44 ± 0.03	0.81	0.93
Temporal	47.91 ± 2.31	49.39 ± 3.03	49.67 ± 2.91	0.07	0.08	0.46 ± 0.05	0.46 ± 0.03	0.46 ± 0.04	0.93	0.89
Whole area	44.50 ± 1.97	44.98 ± 1.85	4,503 ± 2.11	0.08	0.07	0.44 ± 0.04	0.44 ± 0.02	0.45 ± 0.03	0.64	0.61

CP, capillary perfusion; FI, flux index.

## Discussion

In this study, we investigated macular and peripapillary OCTA parameters in PSS patients. Our data showed that although the reduction in IOP was 26.05 ± 7.9 mm Hg (60.3%), neither the papillary nor the macular vessel density measured by OCTA displayed any changes. These findings indicate that patients with PSS might have strong retinal autoregulation to maintain the blood flow in the ONH and macula under remarkably elevated IOP for several days. To our knowledge, this is the first study to investigate the effect of IOP reduction on the retinal microcirculation in patients with PSS.

The autoregulation of blood flow in the eye is the ability to maintain ocular blood flow at a constant level despite alterations in ocular perfusion pressure. Autoregulation of the ocular blood flow in response to the changes in IOP has been investigated in many studies, and most of them have shown that the blood supply to the retina and ONH is well autoregulated. Wang et al. found that the ONH blood flow remained at a constant level within a range of ocular perfusion pressure of 41 mm Hg and above in normal and experimental glaucoma monkeys (Wang et al., 2014). Riva and colleagues found that the blood flow in the optic nerve region remained constant when the IOP was increased to 40 mm Hg by the suction cup (Riva et al., 1997). Conway and coworkers assessed the blood flow responses before and after the application of a microkeratome for refractive corneal surgery (Conway et al., 2010). After elevating the IOP to a level above 85 mm Hg for 90 s in 10 eyes, no differences in ocular perfusion were detected between the baseline measurements and the readings obtained shortly after the IOP elevation. Zhang et al. (2018) used OCTA to investigate changes in the retinal vessel density in ONH and macula after IOP elevation caused by 2 h darkroom prone provocative test; they found that neither the papillary nor the macular vessel density changed markedly after an acute IOP elevation of 10–15 mm Hg for 2 hours. The results obtained in our study are consistent with the aforementioned findings, and autoregulation might explain PSS patients’ response to changes in IOP. More importantly, the baseline IOP was higher, the IOP reduction (26.05 ± 7.9 mm Hg) was larger, and the duration of high IOP was longer in our study. Therefore, our findings strongly indicate that elevated IOP is not the key factor that directly influences retinal perfusion.

Nevertheless, some studies have shown a significant increase in the retinal vessel density after a mild-to-moderate IOP decrease in glaucoma patients as quantified by OCTA. Two studies have shown a significant increase in the peripapillary vessel density 3 months after trabeculectomy, with an average IOP decrease of 14 and 18 mm Hg (Shin et al., 2017; In et al., 2018). Hollo showed that peripapillary vessel density increased in six patients after a medical reduction of IOP of at least 50% from baseline in patients with IOP higher than 35 mm Hg (Holló, 2017). Although these findings support a connection between IOP reduction and increased ocular perfusion, the mechanism needs further elucidation.

An interesting study carried out by Shin et al. (2017) elucidated this mechanism. They found that IOP reduction after trabeculectomy reduced some patients’ LCD. In patients with LCD reduction, microvascular improvement was detected, while in patients without LCD reduction, there was no significant change in retinal microvascularization. Their results indicated that microvascular improvement following trabeculectomy highly correlated with an LCD reduction rather than with an IOP reduction.

Recently, multiple studies have suggested that the vessel density loss occurs later than neural structural changes in glaucoma patients and may be a consequence of a neural structure defect (Lee et al., 2016; Rao et al., 2017a; Rao et al., 2017b; Kim et al., 2017). Si Bum Kim et al. found that the peripapillary vessel density at the area of the RNFL defect in eyes with preperimetric glaucoma was higher than that in perimetric glaucomatous eyes, but it was similar to that in normal eyes (Kim et al., 2017). Harsha et al. compared the primary angle closure (PAC) patients who had a history of high IOP but a healthy disc on clinical exam with control eyes, and they found that the vessel densities in PAC eyes were similar to those of control eyes, although the peripapillary RNFL in the superotemporal sector was significantly thinner in the PAC eyes (Rao et al., 2017a). These results have provided evidence that microvascular changes in the retina occured after RNFL defects. Lee et al. investigated the topographic relationship between the decreased parapapillary retinal microvasculature and the RNFL defect in eyes with primary open-angle glaucoma (POAG), and they found that areas of retinal hypoperfusion on OCTA coincided with the RNFL defect areas (Lee et al., 2016). These authors hypothesized that if decreased perfusion had been the primary driver of glaucomatous optic neuropathy (GON), the areas of the decreased vessel density detected by OCTA would follow the course of the retinal vessel changes; in contrast, if the changes had been the result of GON, decreased vessel density would be observed only in an area of RNFL defect. Therefore, they suggested that decreased VD in glaucoma patients was the result of capillary shutdown secondary to RNFL cell death. Rao et al. (2017b) also found that OCTA had an increased diagnostic ability in eyes with higher baseline IOPs by examining OCTA VD in normal tension glaucoma (NTG) and POAG. They suggested that if perfusion reduction had been the predominant pathogenic mechanism in NTG, eyes with low baseline IOP would have shown a greater difference in VD compared to controls than POAG with high baseline IOP, so they suggested that VD changes were secondary to glaucomatous optic neuropathy. In our study, neither the RNFL loss nor the LC defect was found in PSS patients, and the VF was normal at baseline and at 1-week follow-up. These data supported the fact that the attack of high IOP did not damage the neural structure in our study, which could explain the unchanged retinal blood flow in our PSS patients.

A limitation in our study was that the selected PSS patients displayed no damage in their visual field and the thickness of RNFL. Since some patients with PSS may have optic nerve injury after several high IOP attacks (Megaw and Agarwal, 2017), we should include these patients to elucidate the association between the blood flow changes in the retina and optic neuropathy in future research.

In conclusion, patients with PSS did not show significant changes in the peripapillary and macular capillary vessel density after a marked IOP reduction. The results indicated that elevated IOP is not the key factor that directly influences retinal perfusion in patients with PSS. The defects in the neural structure of these patients, such as LC displacement and RNFL thinning, but not changes in IOP itself, may be responsible for the changes in the retinal blood flow.

## Data Availability

The original contributions presented in the study are included in the article/Supplementary Material; further inquiries can be directed to the corresponding author.
